# Impaired Health-Related Quality of Life in Adolescent Myalgic Encephalomyelitis/Chronic Fatigue Syndrome: The Impact of Core Symptoms

**DOI:** 10.3389/fped.2019.00026

**Published:** 2019-02-15

**Authors:** Maria Roma, Colleen L. Marden, Marissa A. K. Flaherty, Samantha E. Jasion, Erica M. Cranston, Peter C. Rowe

**Affiliations:** Division of General Pediatrics and Adolescent Medicine, Department of Pediatrics, Johns Hopkins University School of Medicine, Baltimore, MD, United States

**Keywords:** myalgic encephalomyelitis, chronic fatigue syndrome, health-related quality of life, orthostatic intolerance, post-exertional malaise

## Abstract

**Objective:** The objectives of this study were to compare the health-related quality of life (HRQOL) of a North American population of adolescents and young adults with myalgic encephalomyelitis/chronic fatigue syndrome (ME/CFS) to (1) healthy controls (HC), (2) adolescents with ME/CFS in other countries, and (3) other forms of pediatric chronic illness, and (4) to examine the influence of the core illness symptoms in the Institute of Medicine (IOM) case definition on impaired HRQOL.

**Study design:** Cross-sectional study comparing individuals with ME/CFS referred to a tertiary care Chronic Fatigue clinic and HC. Eligible participants were age 10–30 years and met the Fukuda criteria for CFS. HC were eligible if they were age 10–30 years, with self-reported good, very good, or excellent general health. Pediatric HRQOL was measured using the PedsQL (Pediatric Quality of Life Inventory) and other validated instruments.

**Results:** We enrolled 55 consecutive ME/CFS patients (46 F) aged 10–23 years. From a pool of 69 potential HC we selected 55 with similar age and gender distribution for comparison. The total and subscale scores on the PedsQL and on all other measures of HRQOL indicated significantly worse function among those with ME/CFS (all *P* < 0.001). The self-reported frequency of post-exertional malaise (PEM) was significantly associated with the severity of impaired HRQOL (*P* < 0.001). Cognitive impairment had a weaker association with the PedsQL score (*P* = 0.02). Orthostatic intolerance was present in 96% of the ME/CFS population. Of the 55 who satisfied the Fukuda criteria, 47 (85%) also satisfied the IOM criteria for the diagnosis. Those meeting the IOM criteria had worse PedsQL total scores than those meeting just the Fukuda criteria (*P* < 0.001).

**Conclusions:** HRQOL was substantially lower in an ambulatory population of adolescents and young adults with ME/CFS than for healthy controls in North America, consistent with reports from other continents. HRQOL was also lower in ME/CFS than has been described in children with asthma, diabetes mellitus, epilepsy, eosinophilic gastroenteritis, and cystic fibrosis. The findings of this study lend further support to the inclusion of PEM, cognitive impairment, and orthostatic intolerance as core symptoms of pediatric ME/CFS.

## Introduction

Myalgic encephalomyelitis/chronic fatigue syndrome (ME/CFS) is a serious, complex, multisystem disorder (1, 2). Regardless of the criteria used to make the diagnosis, ME/CFS is characterized by a substantial impairment in previously tolerated levels of activity (1, 3–7). A relatively small number of studies have compared the health-related quality of life (HRQOL) in pediatric ME/CFS to that of healthy children. These studies have used different ME/CFS case definitions and different measures of overall function (8–11). In this study, we used data collected as part of the Johns Hopkins Pediatric CFS cohort study to compare the HRQOL in our population to healthy controls, to those with ME/CFS in European and Australian samples, and to published results in other pediatric chronic illnesses.

Most research on pediatric ME/CFS has been conducted using either a broad definition of CFS that requires the new onset of three or more months of disabling, unexplained fatigue, (4) or the International Chronic Fatigue Syndrome Study Group criteria for the illness, often termed the Fukuda criteria (3). The Fukuda criteria require at least 6 months of unexplained fatigue, together with the concurrent presence (for at least 6 months) of four of eight symptom criteria (unrefreshing sleep, self-reported impairment in short-term memory or concentration, sore throat, tender cervical or axillary lymph glands, muscle pain, multi-joint pain without joint swelling or redness, headaches of a new type, pattern, or severity, and post-exertional malaise [PEM] lasting more than 24 h). PEM refers to the exacerbation of fatigue but also other symptoms following increased physical or cognitive effort. Data from pediatric ME/CFS studies in the last 25 years have emphasized the frequency and clinical impact of PEM (12–14). In part to reflect the prevalence of PEM in clinical samples, revised expert consensus definitions for the illness have regarded PEM as an essential symptom without which ME/CFS should not be diagnosed (1, 5–7). In the most recent of the case definitions, a committee of the United States Institute of Medicine (IOM) conducted a review of the evidence on major ME/CFS symptoms and manifestations. The committee proposed that the main criteria for the diagnosis should be (1) a substantial reduction or impairment in the ability to engage in pre-illness levels of activity, persisting for more than 6 months and accompanied by new-onset fatigue, (2) post-exertional malaise, (3) unrefreshing sleep, and either (4a) cognitive impairment or (4b) orthostatic intolerance. The IOM committee recommended that the diagnosis of ME/CFS be questioned if these features were not present at least half the time and with at least moderate severity (1).

Operationalizing the IOM criteria requires further work, especially in pediatrics, as children might not be aware that certain symptoms are abnormal, and might not be able to attribute a specific grade of severity to each individual symptom. Because only one pediatric study thus far has examined the IOM criteria in detail (15), two additional objectives of the current investigation were to determine the proportion of study participants who met the Fukuda criteria alone vs. the Fukuda and IOM criteria, and to examine the relationship between overall impairment in HRQOL and the specific core criteria in the IOM definition.

## Methods

### Participants

Consecutive individuals with ME/CFS were included if they had been referred to the Johns Hopkins Children's Center Chronic Fatigue Clinic between October 2008 and December 2012, were age 10–30 years, and satisfied the 1994 International Chronic Fatigue Syndrome Study Group criteria (3). Participants with ME/CFS entered the study with the expectation that they would be followed and treated clinically for 2 years. Individuals with primary depression who were referred by psychiatrists for evaluation of chronic fatigue were excluded, but those who had developed depression sometime after the onset of ME/CFS were included.

A pool of controls was recruited simultaneously with ME/CFS patients during the course of 4 years. Healthy controls (HC) in the same age range were eligible if they reported good, very good, or excellent general health. HC were recruited using information sheets and posted notices in the same pediatric specialty clinic area that houses the Chronic Fatigue Clinic. The majority of recruited controls consisted of the healthy offspring of health professionals employed at Johns Hopkins Children's Center, the friends of those children, and healthy family members and friends of the ME/CFS participants.

Both cases and controls were excluded if they had conditions or treatments expected to interfere with range of motion measurement, which was a separate focus of the study, as described elsewhere (16, 17). Controls were excluded if they had a self-reported condition often associated with chronic fatigue including ME/CFS, postural tachycardia syndrome (POTS), neurally mediated hypotension (NMH), fibromyalgia, recurrent syncope, or other chronic health conditions that can contribute to fatigue. We excluded controls with major depression as measured by a T-score >65 on the Child Depression Inventory (18, 19) or a score >13 on the Beck Depression Inventory (20, 21). The study was approved by the Institutional Review Board of the Johns Hopkins Medical Institutes. Written, informed consent was obtained from participants or their parents as appropriate.

### Study Measures

Participants completed the following questionnaires about their general health at study entry:

1. Pediatric Quality of Life Inventory (PedsQL): The PedsQL is a brief, 23-item, multidimensional child self-report instrument for measuring HRQOL (22). The 23-item assessment examines how much of a problem the child has experienced in the past month with health and activities, feelings, ability to get along with others (which includes social relations, and stamina), and school functioning (cognition, attendance). Responses to each item range from 0 (never) to 4 (almost always). Raw scores are transformed to total scores that range from 0 to 100, with higher scores indicating better quality of life. Five subscales within the PedsQL address physical, emotional, social, school, and psychosocial functioning. The questionnaire is available in age-appropriate formats (Child Report for ages 8–12, Teen Report for 13–18, or Young Adult for 18–24 years). This instrument is reliable, valid and commonly used in pediatric ME/CFS and other pediatric chronic illness populations (10, 11, 23, 24).
2. Functional Disability Inventory (FDI): This one-page, 15-item self-report instrument for children and adolescents asks whether in the past 2 weeks respondents had any physical trouble or difficulty doing specific activities, such as walking up stairs, being at school all day, walking the length of a football field, or going shopping (25). Responses are scored as: 0 = no trouble, 1 = a little trouble, 2 = some trouble, 3 = a lot of trouble, 4 = impossible. The total score ranges from zero (no difficulty with any activity) to 60 (all activities impossible). The FDI has good reliability and validity. It has been used to study a variety of pediatric health problems, including chronic pain and ME/CFS (8, 26, 27).
3. Wood Mental Fatigue Inventory (WMFI): This questionnaire asks subjects to rate the frequency of nine mental fatigue symptoms in the past month on a Likert scale ranging from not at all (0) to very much (4). Higher scores indicate worse cognitive difficulty (28). This measure has been shown to discriminate effectively between ME/CFS patients who are ill and ME/CFS patients who have recovered (29), to correlate with overall well-being in adolescents and adults with ME/CFS (30), and to correlate with the degree of reported brain fog among those with postural tachycardia syndrome (31).
4. Child Depression Inventory (CDI): This 27 item, self-administered instrument measures the mood of the respondent over the preceding 2 weeks (18). The measure assesses behavioral and cognitive signs of depression, applicable to pediatric populations aged 7–17 years. Scores on the 27 items are ranked from 0 (best) to 2 (worst). T-scores of 65 or higher are considered clinically significant (19).
5. Beck Depression Inventory II (BDI): This self-administered, 21-item scale of depression has been validated in adolescents age 13 and older (20). Respondents rank the severity of individual symptoms of depression (including sadness, loss of pleasures, guilty feelings, self-dislike, indecisiveness, loss of energy, concentration difficulty, and fatigue) on a 0–3 scale. Scores of 14–19, 20–28, and 29–63 indicate mild, moderate, and severe depression, respectively (21).
6. PedsQL Multidimensional Fatigue Scale (MFS): This brief, one-page questionnaire measures how much of a problem individuals have had with specific tasks that reflect general fatigue, sleep and rest, and cognitive fatigue, and total fatigue over the preceding month (32). The questionnaire is valid for patients aged 13–18, as well as for college-aged populations (33). Responses on the 18 items range from 0, never a problem, to 4, almost always a problem. As with the PedsQL, the MFS raw scores are transformed to a 0 to 100 scale, with higher scores indicating less fatigue.
7. ME/CFS Symptom Assessment: All participants responded to a study questionnaire that assessed the frequency of Fukuda criteria CFS symptoms as well as lightheadedness in the 2 weeks before study enrollment. Possible responses for the frequency of lightheadedness, fatigue, body aches, joint aches, headaches, or trouble thinking, remembering, or concentrating, were: (a) all day long, every day, (b) several times a day, every day, (c) once or twice a day, every day, (d) several times a week, but not every day, (e) once or twice a week, (f) I haven't had [this symptom]. For the frequency of sore throats and tender glands, possible responses were: (a) every day, (b) more than 5 days but not every day, (c) a few days (2–5), (d) once, or (e) I have not had [this symptom]. For post-exertional malaise (PEM), we focused on physical activity as the trigger, and asked: “In the last 2 weeks, after mild exercise how often have you felt prolonged fatigue or a feeling of illness that lasts longer than 24 h?” Possible responses were (a) 4 or more times, (b) 2 to 3 times, (c) once, (d) never. For unrefreshing sleep, we asked, “In the last 2 weeks, upon awakening after a night's sleep how frequently have you felt refreshed?” Possible responses included (a) all of the time, (b) most of the time, (c) some of the time, (d) none of the time.

### Other Measurement Criteria

#### Onset of ME/CFS

We categorized the type of onset for ME/CFS as abrupt, abrupt on gradual, or gradual. We deemed the onset abrupt if the symptoms emerged over several days in conjunction with an apparent infectious illness or other acute event, abrupt on gradual if individuals had a gradual onset of symptoms together with a marked exacerbation in association with an apparent infectious illness or other acute event, and gradual if there had been no abrupt or acute change at the onset of symptoms.

#### Measurement of IOM Criteria

To operationalize the IOM criterion for a substantial reduction or impairment in the ability to engage in pre-illness levels of activity, we used a frequency of fatigue occurring at least several days per week, and either a PedsQL total score or an FDI score that was >2 SD worse than the mean reported by HC in this study. To operationalize PEM, we used a self-reported frequency of at least once over 2 weeks for prolonged fatigue or the feeling of illness after mild exercise. Unrefreshing sleep had to be present most or all of the time. Cognitive impairment was measured as a self-reported frequency of difficulty thinking, remembering, or concentrating of several times per week or more, or a score on the WMFI or MFS cognitive subscale of >2 SD worse than the mean reported by HC.

Orthostatic intolerance was considered present if (a) self-reported lightheadedness occurred at least several times per week, (b) there was a history of recurrent syncope in the presence of a structurally normal heart, considered consistent with NMH (34) or (c) previous upright tilt testing or a passive standing testing (performed in patients not being treated with medications for orthostatic intolerance) had confirmed the presence of NMH or POTS. Among individuals not previously diagnosed with or under treatment for orthostatic intolerance, we conducted further orthostatic testing using a passive standing test, methods for the performance of which along with study definitions for POTS and NMH are described elsewhere (35).

### Statistical Analysis

We compared the demographic and HRQOL measures between ME/CFS patients and healthy controls using independent samples *t*-tests or Chi-square tests and Fisher's exact tests depending on the type and distribution of the data. Responses on the symptom frequency questions for PEM, lightheadedness, and difficulty thinking and concentrating were trichotomized based on distributions that made clinical sense and created subgroups adequate for statistical comparison. We then compared these trichotomized symptom frequencies to the PedsQL total score or the FDI using one-way ANOVA; any significant differences between groups were then explored further using the *post-hoc* Bonferroni test. Statistical analyses were conducted using IBM Statistics SPSS version 25 (IBM Statistics, New York) and illustrations were prepared using GraphPad Prism version 7.00 for Windows (GraphPad Software, La Jolla, California, USA, www.graphpad.com). The analysis involved multiple comparisons, but not all comparisons were independent. To reduce the probability of a type I error, we considered a *P* < 0.01 as significant.

## Results

### Study Population

Fifty-five consecutive participants who met the 1994 Fukuda criteria for ME/CFS (age range, 10–23 years) were included in this study. Over the study period, we recruited 69 HC. From that group, a research assistant blinded to the quality of life data selected 55 controls who were similar in age and gender to the ME/CFS patients. As shown in Table 1, 95% of the ME/CFS participants were white, and 84% female. Individuals with ME/CFS had been symptomatic for a median of 2 years before entry to the study (range, 7 months to 10 years). All were ambulatory; no participant was primarily bed-bound.

**Table 1:** Demographic and clinical characteristics of the study participants.

	**ME/CFS (*n* = 55)**	**HC (*n* = 55)**	**P**
**DEMOGRAPHIC VARIABLES**			
Age, mean (SD)	16.5 (2.1)	17.1 (3.0)	0.25
Gender			1
Male	9	9	
Female	46	46	
Racial group			0.23
White	52	51	
American Indian	2	0	
Asian/Pacific Islander	1	2	
Other	0	2	
Hispanic			
No	55	52	
Yes	0	3	
**TYPE OF ME/CFS ONSET (*****n*** **=** **55)**			
Abrupt	25		
Gradual	22		
Abrupt on gradual	8		

Twenty-five had developed ME/CFS symptoms abruptly in association with an apparent infectious illness or other acute event. An additional eight had an abrupt increase in the intensity of symptoms against a background of gradual development of some ME/CFS symptoms, while 22 had a gradual onset.

### Prevalence of Fukuda Features

Using cut-points that were closest to symptoms being present at least half the time, Table 2 shows the rank order of the prevalence of Fukuda criteria symptoms for the 55 with ME/CFS and the 55 HC. As expected, those with ME/CFS had greater self-reported prevalence of all symptoms compared to HC. Of note, those with ME/CFS were most likely to report fatigue, unrefreshing sleep, PEM, and cognitive impairment, and were least likely to report sore throat, joint aches, and tender glands. Among controls, 15–20% endorsed unrefreshing sleep, body and joint pain, lightheadedness, and headaches several times per week.

**Table 2:** Comparison of self-reported frequency of Fukuda criteria symptoms in the preceding 2 weeks.

	**ME/CFS *(*n = 55)(%)**	**HC *(*n = 55)(%)**	**P**
**FUKUDA CRITERIA**			
Fatigue (several times/week or more)	100	5	<0.001
Unrefreshing sleep (most/all of the time)	98	18	<0.001
Post-exertional malaise (at least once in 2 weeks)	95	7	<0.001
Cognitive impairment (at least several times/week)	82	2	<0.001
Headache (several times/week or more)	77	18	<0.001
Body pain (several times/week or more)	69	20	<0.001
Sore throat (at least once/week)	51	7	<0.001
Joint aches (several times/week or more)	44	20	0.01
Tender glands (at least once/week)	40	2	<0.001

### HRQOL Comparisons Between ME/CFS Participants and Healthy Controls

**Figure 1:**
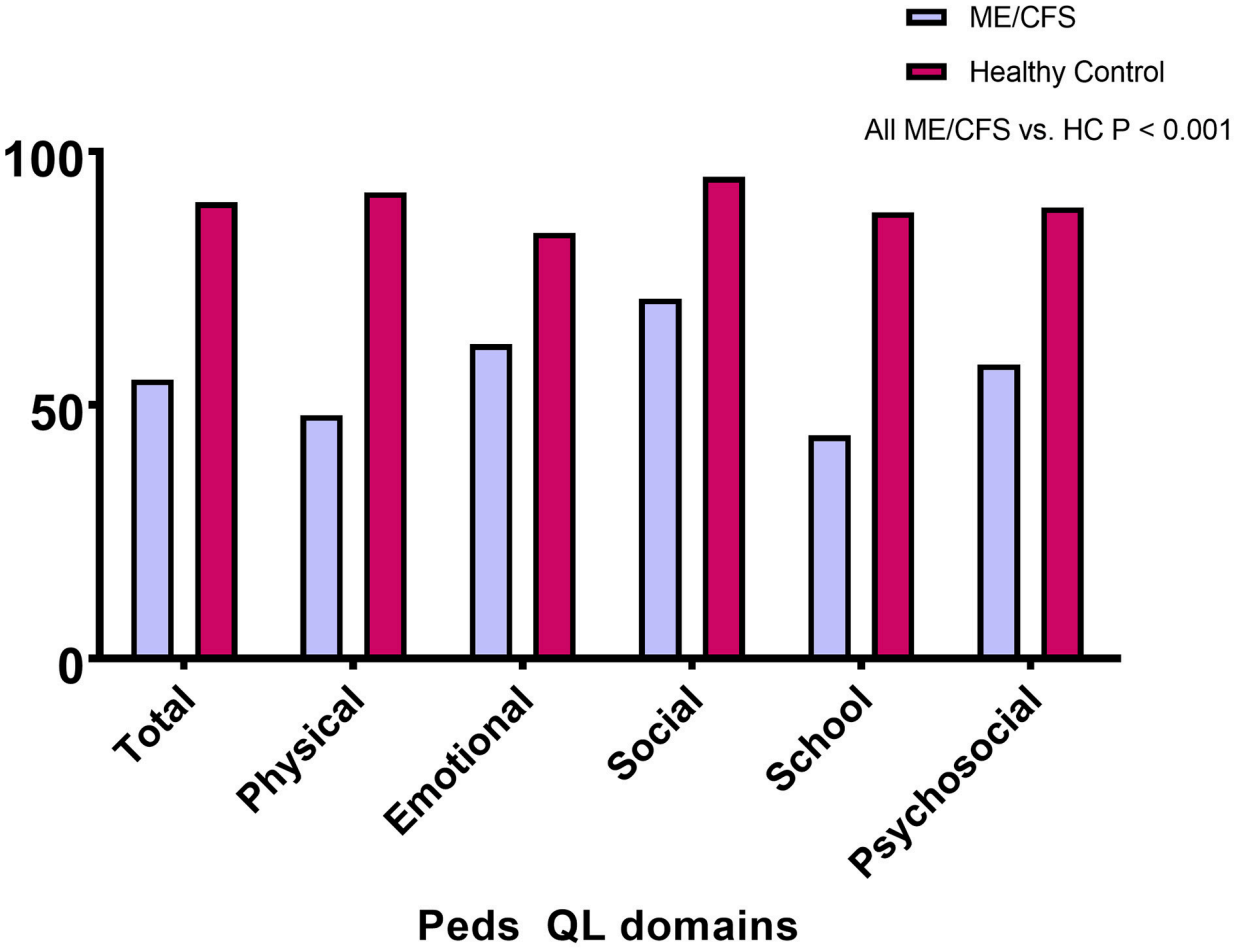
PedsQL total and subscale scores for those with ME/CFS and healthy controls.

Of the 55 with ME/CFS, 21 (38%) had changed from regular schooling due to their symptoms: seven (13%) had switched from full-time to part-time schooling, and 14 (25%) had received home tutoring. Figure 1 shows that the PedsQL total and subscale scores were significantly lower for those with ME/CFS than for healthy individuals (all *P* < 0.001). As displayed in Table 3, the scores on all other measures showed significantly worse HRQOL for those with ME/CFS than for healthy controls (*P* < 0.001).

**Table 3:** HRQOL comparisons for the FDI, MFS, and WMFI scales.

	**ME/CFS *(n = 55)***	**HC *(n = 55)***	***P*-value**
			
FDI	21 (10)	2 (3)	<0.001
**MFS**			
Fatigue total	40 (18)	85 (12)	<0.001
Fatigue cognitive	52 (24)	89 (13)	<0.001
Fatigue sleep	34 (18)	77 (15)	<0.001
Fatigue general	34 (19)	89 (12)	<0.001
WMFI	14 (9)	2 (3)	<0.001

*HC, healthy controls; FDI, Functional Disability Inventory; MFS, PedsQL Multidimensional Fatigue Scale; WMFI, Wood Mental Fatigue Inventory*.

The study design excluded controls if they met criteria for depression, invalidating comparisons with the ME/CFS group. Of those with ME/CFS, 54/55 completed the BDI, the mean (SD) score for which was 15.3 (7.6). Forty-three percent had scores <14, 31% had scores from 14 to 19, 20% had scores from 20 to 28, and 6% had scores of 29 or higher. One child who was too young to complete the BDI had a normal CDI score. There was a significant negative correlation of the BDI with the PedsQL total score (*r* = −0.68; *P* < 0.001). Seven of the 46 (15%) who completed the CDI had T-scores in the clinically significant range of ≥65.

### HRQOL Comparisons With Other Pediatric ME/CFS Studies

Table 4 illustrates the scores for the ME/CFS participants and HC from other studies that use the PedsQL to measure HRQOL in this illness. The PedsQL total score was slightly lower for the study populations from Norway and Australia than for our cohort. The relative pattern of subscale results was similar for those with ME/CFS participants in all studies, identifying relatively lower results for school function, and physical function than for social or emotional function.

**Table 4:** Comparison of PedsQL total and subscale scores with other pediatric ME/CFS studies.

	**Roma ME/CFS *(n = 55)***	Winger^*^ ME/CFS *(n = 120)*	**Knight^‡^ME/CFS *(n = 42)***	**Roma HC *(n = 55)***	**Winger HC *(n = 39)***
Total PedsQL	55 (15)	49 (13)	49 (15)	90 (10)	93 (8)
Physical	48 (17)	37 (17)	42 (23)	92 (9)	96 (8)
Emotional	62 (22)	60 (18)	57 (21)	84 (17)	88 (14)
Social	71 (20)	70 (15)	66 (18)	95 (9)	98 (4)
School	44 (20)	36 (19)	31 (17)	88 (12)	88 (14)
Psychosocial	58 (17)	57 (15)	51 (14)	89 (12)	91 (10)

* *Data from Winger et al. (10)*.

‡ *Data from Knight et al. (11)*.

The mean *(SD)* FDI score in this study was 21 (10), which was similar to the means of 24.0 (9.2) and 23.1 (9.2) for the 60 Norwegian ME/CFS adolescents in each group randomized to clonidine or placebo, respectively (27). Among 68 HC in that study, the mean FDI score was 1.6 (3.1). These results were also similar to the mean score of 24 in a sample of 20 with ME/CFS reported from the UK (8).

### Relationship of PEM, Cognitive Impairment, and Lightheadedness to HRQOL

**Figure 2:**
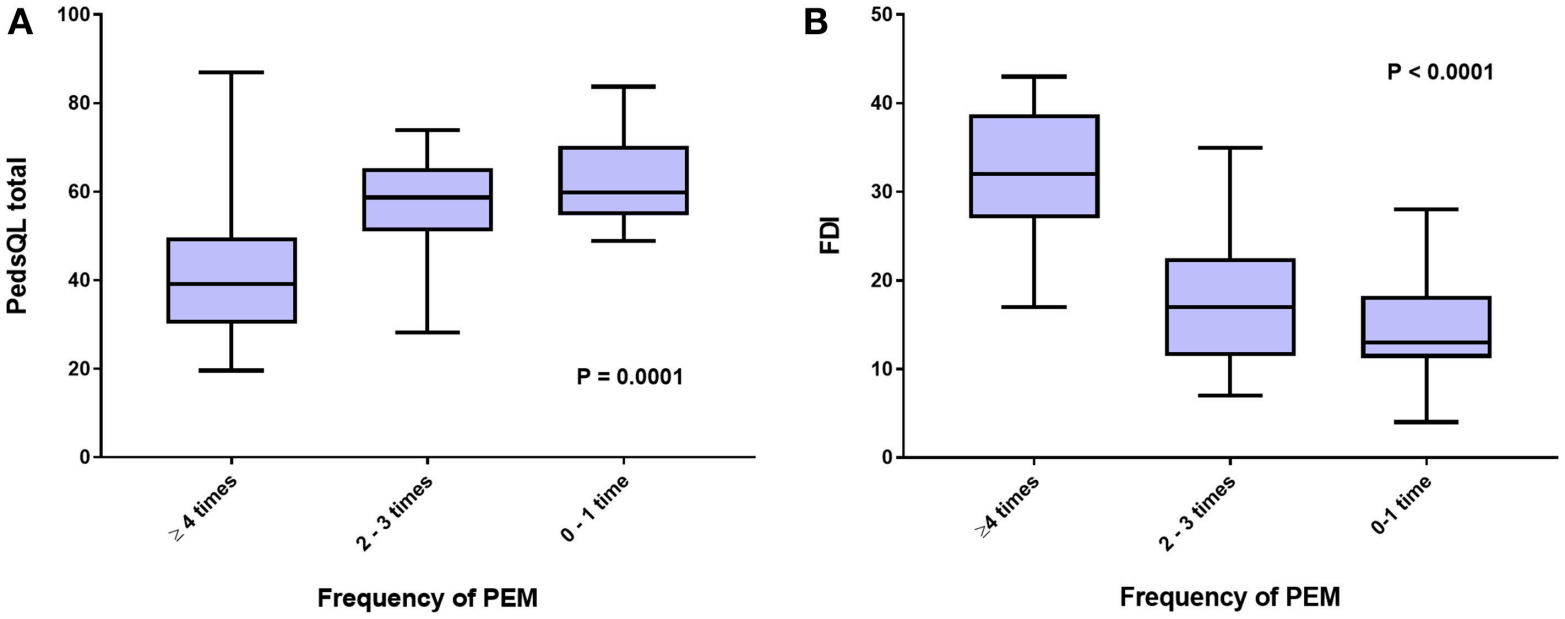
HRQOL measured by **(A)**, Peds QL total score, and **(B)**, FDI, according to self-reported frequency of post-exertional malaise (PEM) in the preceding 2 weeks.

To further investigate the interaction between the proposed core ME/CFS criteria and HRQOL, we examined whether greater frequencies of PEM, cognitive impairment, and lightheadedness were associated with worse scores on the various measures. Figure 2A shows that those reporting more frequent PEM in the preceding 2 weeks had significantly lower scores on the PedsQL (ANOVA F score = 10.73; *P* = 0.0001). *Post-hoc* comparisons using the Bonferroni test indicated that PedsQL scores were lower in those reporting PEM at least four times in 2 weeks than those reporting PEM 0-1 time (*P* < 0.001) or 2–3 times (*P* = 0.001), but not significantly different between those reporting PEM 2–3 times and 0–1 time (*P* = 0.78).

A significant association was also present for the relationship between the frequency of PEM in the past 2 weeks and scores on the FDI (Figure 2B) (ANOVA F score = 26.1; *P* < 0.0001). *Post-hoc* comparisons using the Bonferroni test indicated that FDI score was lower in those reporting PEM at least four times in 2 weeks than those reporting PEM 0–1 time (*P* < 0.001) or 2–3 times (*P* < 0.001), but not significantly different between those reporting PEM 2–3 times and 0–1 time (*P* = 0.18).

**Figure 3:**
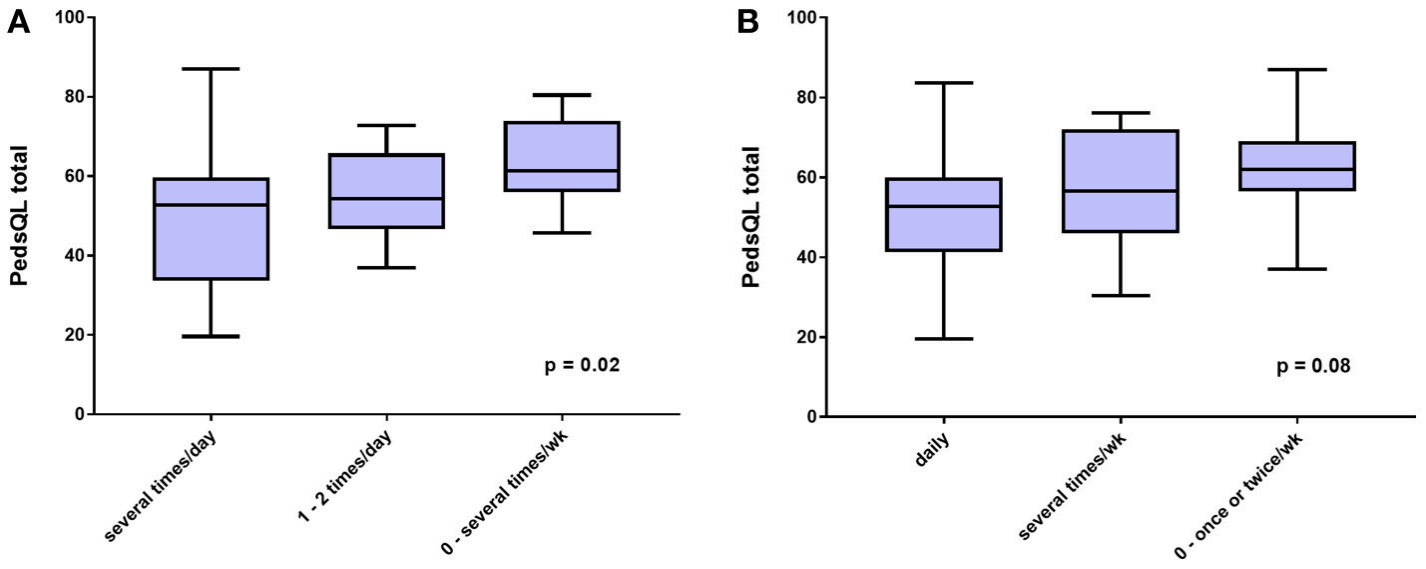
HRQOL measured by PedsQL total score according to **(A)**, self-reported frequency of trouble thinking and concentrating, and **(B)**, self-reported frequency of lightheadedness, both in the preceding 2 weeks.

As shown in Figure 3A, the relationship of cognitive impairment with the PedsQL total score was weaker (ANOVA F score = 4.55; *P* = 0.02), as was the relation between the frequency of lightheadedness and the PedsQL total score (Figure 3B; ANOVA F score = 2.72; *P* = 0.08).

### Measuring the IOM Criteria

Table 5 shows the prevalence of impaired function, unrefreshing sleep, PEM, cognitive impairment, and orthostatic intolerance according to the methods we used for operationalizing the IOM criteria. Along with self-reported frequency of unrefreshing sleep, PEM, and the other core symptoms, we defined a substantial impairment in function as a questionnaire score that was 2 SD worse than the mean of healthy controls for the PedsQL (scores of 70 or below) or the FDI (scores of eight or higher). To supplement the self-reported frequency of trouble thinking and concentrating, we also used score of 2 SD worse than the mean for healthy controls on the WMFI (scores >8) or the MFS (scores of <63).

**Table 5:** Prevalence of Institute of Medicine ME/CFS criteria.

	**ME/CFS (*n* = 55)(%)**	**HC (*n* = 55)(%)**	***P***
1. Substantially impaired function
Fatigue (several times/week or
more)	100	5	<0.001
FDI or PedsQL >2 SD worse
than mean scores for HC^*^	96	7	
2. Unrefreshing sleep (most/all of
the time)	98	18	<0.001
3. Post-exertional malaise (at
least once in 2 weeks)	95	7	<0.001
4.a. Cognitive impairment
Trouble thinking and
concentrating (several
times/week or more)	82	2	<0.001
Symptom frequency plus
WMFI or
MFS cognitive subscale >2
SD worse than mean for HC^*^	93	11	
4.b. Orthostatic intolerance
LH (several times/week or
more)	76	15	<0.001
LH frequency plus pre-study
diagnosis/treatment of OI	84	15	<0.001

* *P-values not computed because of the >2SD method of operationalizing the criterion*.

*HC, healthy controls; FDI, Functional Disability Inventory; PedsQL, Pediatric Quality of Life Inventory; WMFI, Wood Mental Fatigue Inventory; MFS, PedsQL Multidimensional Fatigue Scale; LH, lightheadedness; OI, orthostatic intolerance*.

Of those with ME/CFS, 82% reported a frequency of cognitive impairment (difficulty thinking and concentrating) of at least several times per week vs. just 2% of healthy controls. Combining the self-reported symptom frequency with a WMFI or MFS cognitive subscale >2 SD worse than the mean for healthy controls identified six additional ME/CFS participants and five additional controls as meeting the criteria for trouble thinking and concentrating. As expected, there was a strong negative correlation between the WMFI scores and the cognitive subscale of the PedsQL MFS (*r* = −0.90; *P* < 0.001).

Lightheadedness at least several times per week was endorsed by 42/55 (76%) of those with ME/CFS. Four others who reported lightheadedness 1–2 times per week or less met other criteria for orthostatic intolerance: two had been diagnosed with NMH before study entry and were being treated with fludrocortisone at the time of the symptom questionnaire, and two others had a history of recurrent syncope in the presence of a structurally normal heart and were being treated with an increased intake of sodium and fluids at the time of questionnaire completion. Among the nine remaining participants with ME/CFS whose questionnaire responses indicated a frequency of lightheadedness of 1–2 times per week or less, one adolescent reported a discrepant history during the clinical interview, describing a daily “head rush” (identical to lightheadedness) with postural changes. She was therefore re-classified. Thus, 84% met the study criteria for orthostatic intolerance using the criteria of frequent lightheadedness and a prior diagnosis or treatment of orthostatic intolerance.

The remaining eight participants underwent a 10 min passive standing test; four were receiving no vasoactive medications at the time of testing, and four were being treated with one or more medications that had some potential to affect the HR and BP responses to orthostatic testing (amitriptyline for headaches [*n* = 1], stimulant medications for attention deficit disorder [*n* = 1], selective serotonin or serotonin/norepinephrine reuptake inhibitors for anxiety or low mood [*n* = 2], and oral contraceptives [*n* = 1]). Of the four medication-naïve participants, two developed POTS during the 10-min standing test, each with a 50 beat per min increase in HR while upright, and a third became presyncopal at the 6-min point of upright posture, consistent with NMH. All four had worsening or provocation of their typical chronic orthostatic or ME/CFS symptoms during the period of standing (most commonly increased fatigue, lightheadedness, and warmth). Of the four who were being treated with vasoactive medications, three developed POTS and increased symptoms, and the fourth reported increased orthostatic symptoms only during the passive standing test. In all, 96% had evidence of orthostatic intolerance if those who developed POTS or NMH during the standing test were added.

### Proportion Satisfying the IOM Criteria

Forty-seven of the 55 participants (85%) who met the Fukuda criteria also satisfied the IOM criteria. Although all who met the Fukuda criteria reported fatigue several times per week or more, and all had some reduction of their pre-illness activity levels, when we applied the formal study criteria for a substantial reduction, two participants did not have a PedsQL or FDI score more than 2 SD below the mean of the healthy controls. A third participant reported refreshing sleep most of the time. Three further individuals did not endorse PEM. Two additional participants met all other criteria except for cognitive impairment or the study criteria for orthostatic intolerance. These eight who satisfied the Fukuda criteria but not the IOM criteria had significantly better scores than the 47 who met both the Fukuda and the IOM criteria on the PedsQL [70 (7) vs. 53 (15), *P* < 0.001], and on the MFS [59 (15) vs. 36 (17), *P* = 0.001]. There were marginally non-significant differences on the FDI [13 (7) vs. 22 (10), P = 0.01], the WMFI [8 (7) vs. 15 (9), *P* = 0.03], and the BDI [10 (6) vs. 16 (7); *P* = 0.03].

## Discussion

### HRQOL in ME/CFS

The results of this study provide further confirmation of earlier reports that adolescents and young adults with ME/CFS have a significantly lower HRQOL than their healthy peers. The mean PedsQL total score in this North American population is similar to the results reported by Knight et al. in Australia using the Fukuda criteria and by Winger et al. in Norway using a broad case definition (10, 11). The scores on the FDI were also similar to findings from the large study of 120 adolescents in Norway (27) and a small study of 20 in the United Kingdom (8), both of which employed a broad case definition. Although the participants in our study had slightly better overall function as measured by the total PedsQL scores at enrollment, there was a consistency in the distribution of PedsQL subscale scores across the three studies using that measure: all reported less impairment in the social and emotional domains of the PedsQL, and greater impairment in the domains measuring physical function and school function. The low scores on HRQOL confirm the findings of Kennedy et al. using the Child Health Questionnaire to assess 25 UK children who met the Fukuda criteria. In that study, scores on limitations due to physical health problems were lower than scores reporting limitations due to emotional or behavioral difficulties (9).

One objective of the study was to situate the HRQOL results for those with ME/CFS within the context of other chronic pediatric health conditions. The PedsQL has been used to measure overall function in many chronic pediatric illnesses, allowing comparison across diagnoses (23, 24). The mean (SD) PedsQL total score for those with ME/CFS in our study [55 (16)] was lower than the reported scores for North American children with cystic fibrosis [80 (14)], eosinophilic gastrointestinal disorder [68 (14)], epilepsy [76 (14)], type 1 diabetes [74 (16)], sickle cell disease [70 (18)], and renal transplants [75 (15)] (24), and comparable to pediatric fibromyalgia [56 (16)] and diplegic cerebral palsy [54 (13)] (23). One methodological caveat is that the PedsQL scores were not obtained at the same point of treatment for all chronic conditions, so direct statistical comparisons would be misleading. Treatment of ME/CFS had been initiated in only some of our patients at the time of administration of the PedsQL, whereas clinical samples of patients in the other studies likely would have included individuals at different stages of the usual treatment of their conditions, therefore resulting in higher scores than at the outset of treatment. Nonetheless, even without incorporating the scores of severely impaired ME/CFS patients, who were unable to participate in clinic-based studies, the lower scores of the ME/CFS participants emphasize the profound degree of interference of the illness with normal activities in childhood.

A novel finding of this study is the correlation of impairment in HRQOL with the frequency of PEM, at least for an ambulatory population with ME/CFS. Prior investigations of the importance of PEM had compared its frequency in those with ME/CFS to healthy controls, but, surprisingly, to the best of our knowledge, no study had examined the association of PEM with the severity of impairment or HRQOL, or with the severity of specific symptoms. Such comparisons would be important as a test of whether PEM is a critical symptom that should be accounted for in illness definitions. In our study, as the frequency of PEM increased, the mean PedsQL score fell and the mean FDI score increased, consistent with a significant association of PEM with worse overall function. This relationship might not be expected to obtain for those who are bedbound, as they might be too ill to engage in much physical activity or might electively restrict their activities to avoid provoking this symptom. Although a limitation of our assessment of PEM was that we only asked about PEM induced by mild physical activity, the responses to this single question identified PEM in 95% of the study population. Future studies will need to examine the added yield of questions about PEM following varying degrees of cognitive, orthostatic, or neuromuscular stress (36–38).

### Implications for Measuring Orthostatic Intolerance

An important aspect of the study methodology was that we included a comprehensive evaluation of orthostatic intolerance, incorporating the self-reported frequency of lightheadedness as well as a detailed clinical history and, where necessary, orthostatic testing. Although significantly more ME/CFS participants described lightheadedness several times per week or more compared to healthy controls [76 vs. 15%, *P* < 0.001], the prevalence of orthostatic intolerance increased to 84% when a history of recurrent syncope or prior positive orthostatic testing was included. A further 12% developed POTS or NMH in response to a 10-min passive standing test in clinic, all of whom reported provocation of their usual ME/CFS symptoms when standing. There is as yet no consensus on which of these criteria should be considered valid for confirming orthostatic intolerance. Among the caveats about orthostatic testing that need to be considered are that (1) the response to head-up tilt table testing can be abnormal in otherwise healthy individuals, some of whom develop hypotension or syncope during the procedure (30, 39), (2) as the current HR criteria for POTS are defined, approximately 5% of healthy adolescents would have at least a 40 bpm increase in heart rate during 10 min upright (39), some of whom might endorse chronic lightheadedness in daily life. We are unsure how many healthy individuals would develop increased orthostatic symptoms during a 10-min passive standing test, although the available data from tilt testing suggests this would be infrequent. Singer et al. reported that 8/106 (8%) of healthy controls developed orthostatic symptoms during a 10 min head-up tilt test, none of whom had a history of syncope or orthostatic symptoms (39). Moreover, chronic fatigue was reported by only 5% of the healthy controls in our study. Because healthy controls have a low prevalence of chronic orthostatic symptoms in daily life, and a low prevalence of orthostatic symptoms provoked during upright tilt, we would assume a similarly low rate of provocation of orthostatic symptoms in healthy adolescents during passive standing tests. These caveats notwithstanding, our results illustrate the potential for the prevalence of orthostatic intolerance to be underestimated if it is only measured by self-reported lightheadedness. Other questions that ask about orthostatic provocation of fatigue, PEM, difficulty thinking and concentrating, headache, pain, nausea, and warmth deserve further study to determine whether they add to the yield of lightheadedness as a reflection of orthostatic intolerance in ME/CFS. Taken together, the findings from this study provide further support for the inclusion of orthostatic intolerance in the case definition of ME/CFS, as was recommended in the IOM report.

### Implications for ME/CFS Case Definitions

Our study findings have several implications for case definitions of pediatric ME/CFS. First, although the 1994 Fukuda criteria for CFS had included post-exertional malaise (PEM) as one of eight symptom criteria, four of which were needed to meet the illness definition, individuals could satisfy the Fukuda criteria without experiencing PEM. Subsequent ME/CFS case series have shown that PEM is described by 71–96% of adolescents with ME/CFS (12–14). The data from our study showing a significant correlation of PEM with overall HRQOL impairment provide further support for the inclusion of PEM in pediatric case definitions for the disorder.

Second, because the Fukuda criteria were published in 1994, and modern attention was only drawn to orthostatic intolerance as a common co-morbid problem in 1995 (30, 40), the Fukuda criteria did not mention lightheadedness as a qualifying diagnostic feature. While orthostatic intolerance is mentioned more explicitly in subsequent case definitions, the Canadian Consensus Criteria (CCC) did not require orthostatic intolerance to be present in order to satisfy the diagnosis in either adults or children (5, 6). The CCC require that individuals report one symptom from among two of the following three categories: autonomic, neuroendocrine, and immune manifestations. It would thus be possible to meet the CCC ME/CFS definition with “recurrent feelings of feverishness and cold extremities” (neuroendocrine) and “sore throat” (immune), without having lightheadedness or objective evidence of orthostatic intolerance. To qualify as having ME/CFS by meeting such vague and non-specific symptom criteria could lead to inclusion of conditions other than ME/CFS in studies. It remains to be seen whether the other qualifying criteria for the pediatric CCC (fatigue for at least 3 months, unrefreshing sleep, pain, and neurocognitive manifestations) capture the main features of the illness well-enough to make the inclusion of the CCC autonomic/neuroendocrine/immune criteria irrelevant, raising the question of whether the diagnostic criteria could be further simplified.

Second, the international ME criteria list orthostatic intolerance as one manifestation under the rubric of “energy production/transportation impairments.” While impaired energy production could be a cause of orthostatic intolerance, impaired energy production is more likely to be a consequence of orthostatic intolerance, directly related to reduced blood flow, and reduced oxygen delivery to tissues. The international ME criteria require only one symptom or feature from among four categories that include cardiovascular, respiratory, loss of thermostatic stability, or intolerance of extremes of temperature. An individual could thus satisfy the ME criteria with intolerance of cold temperature and sweating episodes, without clear evidence of orthostatic intolerance. Given the high prevalence of orthostatic intolerance in pediatric ME/CFS, our data suggest that this symptom requires greater emphasis than it received in the CCC and ME criteria, at least for pediatric case definitions.

Third, the desire for clinicians to have a sensitive case definition that will identify the largest number of individuals (who might then benefit from treatment) conflicts with the desire for researchers to have a more specific, restrictive case definition that identifies those with more profound impairment and ostensibly a more “pure” form of the illness (1). This dilemma is complicated by the lack of scientific clarity regarding whether ME/CFS is a single, unitary condition, or a disorder that encompasses several overlapping conditions with multiple causes. Debates about these issues are unlikely to be resolved until a gold standard diagnostic test is developed. In the current study, the Fukuda criteria identified a slightly larger group than the IOM criteria. Those who met both the Fukuda and the IOM criteria had more severe impairment than those who met the Fukuda criteria alone. Our study proportion meeting the IOM criteria differs substantially from the proportion reported by Asprusten et al. In contrast to the 85% meeting IOM criteria in our study, only 45/114 (39%) of their participants who met the broad definition of pediatric CFS (3 months of unexplained fatigue that interfered with normal school attendance) satisfied the IOM criteria (15). The IOM positive and negative groups in the Asprusten study did not differ on baseline measures except for the depressive symptoms scores on the Mood and Feelings Questionnaire. The FDI and Peds QL scores for the IOM positive and negative groups were not reported; scores on both questionnaires for the overall group were comparable to our study results. Given the similarity in overall HRQOL in participants enrolled in each study, the magnitude of the difference in the prevalence of IOM-positive participants in our study and the Asprusten study is unlikely to be due to chance. Rather, the large difference in proportions meeting the IOM criteria suggest that variability in the methods for operationalizing the IOM definition were responsible for the differences. Our operational definition of PEM required that it occur at least once over 2 weeks, which was reported by only 7% of HC. We assumed that some patients might be restricting their activity sufficiently to avoid provoking PEM, and reasoned that a single episode of prolonged PEM confirmed the presence of that symptom. This might have resulted in a less restrictive criterion for PEM than in the Asprusten study, which required the endorsement of four separate questions on the symptom, illustrating the potential for marked variability in outcomes to result from differences in the way in which disease criteria are operationalized.

Fourth, as the level of functional impairment increased in our participants with ME/CFS, so did the scores on the Beck Depression Inventory. There was a strong negative correlation between the Peds QL and the BDI scores. This result is influenced by the ascertainment in the BDI of several somatic symptoms that would be consistent with ME/CFS. Even among individuals who are free of self-reproach, anhedonia, and depressed mood, respondents could score a up to 15 points on the BDI for items related to indecisiveness, concentration difficulty (cognitive impairment), loss of energy, changes in sleeping pattern, and tiredness or fatigue (fatigue, impaired function), all of which would be expected to be common in adolescents with ME/CFS. We did not measure the independent influence of ME/CFS and mood disorders on HRQOL, but other pediatric investigators have done so. In their population of adolescents with ME/CFS, Winger and colleagues found higher rates of depressed mood using the Mood and Feelings Questionnaire (10). Higher levels of depressive symptoms were associated with lower quality of life in both ME/CFS patients *and* healthy controls, but both depressive symptoms and having ME/CFS were independently associated with worse HRQOL (10). The BDI and other depression questionnaires can be helpful clinically to identify those with greater reporting of self-reproach, guilt, and lack of self-worth—all symptoms that would warrant a greater focus on the individual's affective response to chronic illness. However, the questionnaires cannot distinguish whether the depression preceded or was a secondary reaction to being chronically ill with ME/CFS. Other measures of depressed mood that rely less on overlapping somatic symptoms of ME/CFS deserve further attention in pediatric ME/CFS research, as has been suggested in the adult ME/CFS population (41). The Hospital Anxiety and Depression Scale, as used by other ME/CFS groups (11, 42) might demonstrate less co-linearity and be more appropriate for determining whether there is a true correlation of disease severity and affective state.

## Limitations

This study had several limitations. Because patients were evaluated at a tertiary care center, participants might not be representative of the entire population with pediatric ME/CFS. Our study population would have excluded those at the severe end of the spectrum who were bed-bound and unable to attend frequent clinic visits. Inclusion of bed-bound individuals would have had the effect of further lowering the HRQOL scores. Conversely, we included some with mild ME/CFS who did not meet the more stringent IOM criteria for the illness, although all had some limitation in their overall ability to engage in pre-illness activities. The 2-year median duration of illness and the intensity of the pre-study treatment trials may have affected the scores of HRQOL measurements.

We cannot exclude the possibility of selection and referral biases. For example, our group's interest in orthostatic intolerance might have resulted in a greater referral of individuals suspected of having orthostatic intolerance; 33% had been tested for that diagnosis at enrollment. For the remainder, the clinical history clarified that most were unaware of the relationship of orthostatic intolerance to ME/CFS, and were not aware of the clinical features of POTS and NMH. Other studies that use the same ascertainment methods for orthostatic intolerance will be needed to confirm our results.

The use of a cut-off that was more than two SD worse than the mean scores for HC as the definition of substantial impairment in general function or of cognitive impairment is relatively conservative. Further study will be needed to determine whether this excludes too many who might meet ME/CFS disease criteria using other methods of operationalizing the illness, such as less stringent cut-points of 1.5 SD from the control means.

## Conclusion

The current study confirms a marked impairment in HRQOL in North American adolescents and young adults compared to healthy controls. The HRQOL data were similar to those reported in European and Australian pediatric ME/CFS populations, regardless of which case definition is used. All studies of adolescents report substantially worse function than have been reported for children with other common chronic health impairments. Our study identified a strong correlation of overall HRQOL with the frequency of PEM as well as a >90% prevalence of cognitive impairment and orthostatic intolerance. Individuals who met both the IOM and Fukuda criteria for the diagnosis had worse HRQOL than those who met the Fukuda criteria alone. The data from this study lend further support to the inclusion of PEM, cognitive impairment, and orthostatic intolerance as core features of pediatric ME/CFS, and should help inform future discussions regarding a pediatric case definition.

## Data Availability

The raw data supporting the conclusions of this manuscript will be made available by the authors, without undue reservation, to any qualified researcher.

## Author Contributions

CM and PR designed the study. CM maintained the study database. SJ, MF, EC, and CM contributed to data entry. MR wrote the first draft of the manuscript. All authors contributed to manuscript revision, read and approved of the final manuscript, and data analysis.

### Conflict of Interest Statement

The authors declare that the research was conducted in the absence of any commercial or financial relationships that could be construed as a potential conflict of interest.
